# Effect of dexmedetomidine on postoperative nausea and vomiting in female patients undergoing radical thoracoscopic lung cancer resection

**DOI:** 10.3389/fphar.2024.1353620

**Published:** 2024-01-25

**Authors:** Haipeng Zhu, Shichao Wang, Ruohan Wang, Bing Li, Jiaqiang Zhang, Wei Zhang

**Affiliations:** ^1^ Department of Anesthesiology and Perioperative Medicine, Zhengzhou University People’s Hospital, Henan Provincial People’s Hospital, Zhengzhou, Henan, China; ^2^ Department of Anesthesiology, Binzhou Medical University Hospital, Binzhou, Shandong, China

**Keywords:** female, postoperative nausea and vomiting, thoracoscopic surgery, dexmedetomidine, lung cancer

## Abstract

**Introduction:** Postoperative nausea and vomiting (PONV) is a prevalent postsurgical complication. The objective of our study was to compare the effect of different doses of dexmedetomidine on PONV in female patients undergoing radical thoracoscopic lung cancer resection.

**Methods:** A total of 164 female patients undergoing elective thoracoscopic radical lung cancer surgery were enrolled and assigned to one of four groups. Patients received 0.2 μg/kg/h, 0.4 μg/kg/h, 0.8 μg/kg/h dexmedetomidine and normal saline in the Dex1, Dex2, Dex3 and Control groups, respectively. The primary outcome was the incidence of PONV during 48 h postoperatively. The second outcomes included the incidence of PONV and postoperative vomiting (POV) at four time points postoperatively (T1: PACU retention period; T2: PACU discharge to postoperative 12 h; T3: postoperative 12 h-postoperative 24 h; T4: postoperative 24 h-postoperative 48 h), the area under the curve of PONV grade (PONV_AUC_), PONV grade, POV grade and other postoperative recovery indicators.

**Results:** The incidence of PONV differed among the four groups. The Dex2 group (29.27%) was lower than that in the Dex1 group (61.90%) and Control group (72.50%). The incidence of PONV at T2 in the Dex1 group (11.90%) and Dex2 group (9.76%) was lower than that in the Control group (42.50%). The incidence of PONV at T3 in the Dex2 group (29.27%) was lower than that in the Dex1 group (61.90%) and Control group (62.50%). The PONV_AUC_ was lower in the Dex2 group than in the Control group. The incidence of POV at T3 in the Dex2 and Dex3 groups was lower than that in the Control group. The consumption of remifentanil, norepinephrine, PACU dwell time, VAS scores, postoperative PCA press frequency, and the time for the first postoperative oral intake were different among the four groups. The regression model shows that the Dex2 group is a protective factor for PONV.

**Conclusion:** Dexmedetomidine can reduce the incidence of PONV and accelerate postoperative recovery in female patients undergoing radical thoracoscopic lung cancer resection. Compared with the other two dosages, 0.4 μg/kg/h dexmedetomidine is preferable.

**Clinical Trial Registration:** chictr.org.cn, identifier ChiCTR2300071831

## 1 Introduction

Nausea and vomiting are common gastrointestinal discomforts. Nausea is the unpleasant sensation of having the urge to vomit, whereas vomiting is a physical event and is the forceful expulsion of intestinal and gastric contents through the mouth (Zhong et al., 2021). From the beginning of the use of general anesthesia in the 1840s, it was recognized that postoperative nausea and vomiting (PONV) is a common side effect after surgery (Horn et al., 2014). PONV is an unpleasant experience that affects 20%–30% of surgical patients after general anesthesia, and in patients with high-risk factors, the incidence of PONV in the absence of preventive measures is as high as 80% (Eberhart et al., 2000; Apfel et al., 2012a). PONV decreases patient comfort and satisfaction and may cause dehydration and electrolyte imbalances, aspiration of gastric contents, esophageal rupture, suture dehiscence, and bleeding (Visser et al., 2001; Veiga-Gil et al., 2017).

The mechanism of PONV is complex, and there are many influencing factors. Being female, nonsmoking, having a history of motion sickness, having a history of PONV and taking opioids are regarded as risk factors (Apfel et al., 2012b). In addition, the type of surgery, duration of anesthesia, use of volatile anesthetics, nitrous oxide, and age under 50 years are also important factors related to PONV(Horn et al., 2014).

Thoracic surgery has the characteristics of a strong stress response and systemic inflammatory response, unstable circulation, a high risk of postoperative pulmonary complications, and a high demand for opioid drugs (Huang et al., 2017). With the development of minimally invasive technology, thoracoscopic surgery, which has the advantages of less trauma and fast recovery, has now become the most common surgical method in thoracic surgery. However, the incidence of PONV after thoracoscopic surgery remains high (Zha et al., 2021; Sertcakacilar et al., 2022; Vijitpavan et al., 2022). Vijitpavan’s study showed that the incidence of PONV within 48 h after thoracoscopy was 68.42%–73.68%, and 57.89% of patients needed drug intervention treatment (Vijitpavan et al., 2022).

Researchers have tried various methods to prevent and treat PONV. Among them, medication, such as 5-hydroxytryptamine (5-HT3) receptor antagonists, dopamine receptor antagonists, glucocorticoids, and anticholinergic drugs, is one of the important methods (Gan et al., 2020). However, due to the different pharmacokinetics, efficacy, and side effects of these drugs, as well as controversies regarding the route of administration and dosage, it is necessary to find suitable medications for better prevention and treatment of PONV.

Dexmedetomidine (Dex) is a highly selective α-2 adrenergic receptor agonist (Afonso and Reis, 2012). Dex possesses analgesic, anxiolytic, sympatholytic, organ-protective, and sedative properties similar to natural sleep, and it is now widely used in clinical practice (Chima et al., 2022). The effect of Dex in preventing PONV has been continuously investigated. On the one hand, Dex exerts analgesic effects and reduces the perioperative use of opioid drugs, thereby decreasing the incidence of PONV caused by postoperative pain and opioid use (Bao and Tang, 2020; Tian et al., 2022). On the other hand, Dex has been found to inhibit the release of norepinephrine in the locus coeruleus and reduce the release of 5-HT3 in the median raphe nucleus and dorsal raphe nucleus, thereby reducing the incidence of PONV(Hopwood and Stamford, 2001; Sugino et al., 2021). Wang’s study demonstrated that for adult lung cancer patients undergoing lobectomy, the intravenous infusion of Dex can reduce the incidence of PONV and decrease the use of opioid drugs (Wang et al., 2016). Other studies have shown that the intraoperative use of Dex can reduce the pain visual analog scale (VAS) scores within 48 h after cholecystectomy and orthopedic surgery and decrease the use of postoperative antiemetic drugs and opioids, thereby accelerating patient surgical recovery (Shin et al., 2019; Choi et al., 2021). However, previous studies have the following limitations: 1. No studies have specifically focused on adult female patients who are at high risk for PONV. The effects of Dex on adult females are still unclear. 2. The dosage range of Dex in previous studies has been highly variable, and the appropriate dosage for treating PONV remains unclear. 3. The incidence of PONV may vary depending on the type of surgery. To date, few studies have focused on radical thoracoscopic lung cancer resection, which accounts for an important proportion of thoracic surgeries.

Based on the above considerations, this study intends to observe the effects of different doses of dexmedetomidine on PONV in female patients undergoing thoracoscopic radical lung cancer surgery. We aimed to clarify its clinical efficacy and explore its optimal dose, providing evidence for reducing the incidence of PONV in thoracic surgery patients.

## 2 Materials and methods

### 2.1 Study subjects

This prospective, double-blind, single-center study was performed in accordance with the Declaration of Helsinki, approved by the Institutional Review Board of the Henan Provincial People’s Hospital (Approval number: 2020 lunshen 100), and registered with the Chinese Clinical Trial Registry at www.chictr.org (registration number: ChiCTR2300071831). With written consent, all patients were enrolled between May 2023 and July 2023.

All patients undergoing elective thoracoscopic radical lung cancer surgery were considered for enrollment. The following were the inclusion criteria: scheduled for thoracoscopic radical surgery for lung cancer under general anesthesia; female; aged 18–65 years old; and American Society of Anesthesiology (ASA) Physical Status I-II. Patients who met any of the following criteria were excluded: history of thoracic surgery; use of opioids, antiemetics, or corticosteroids within 1 month before surgery; preoperative chemoradiotherapy; severe cardiovascular and cerebrovascular diseases; severe hepatic or kidney dysfunction; mental disorders; and inability to cooperate to complete follow-up. Patients with intraoperative blood transfusion, postoperative chemoradiotherapy, postoperative ICU transfer, loss to follow-up, withdrawal from the study, and surgical changes were excluded from the final analysis.

### 2.2 Procedure

All patients were treated with total intravenous anesthesia. An anesthesiology nurse who was blind to the study prepared the study agents in identical 50-mL syringes according to the treatment groups, with the treatment group information contained in sequentially numbered sealed envelopes. The surgeon, the anesthesiologist, the follow-up personnel, the statisticians and the patients were blinded to the treatment group.

Electrocardiography, heart rate, invasive blood pressure and pulse oxygen saturation were initiated upon arrival in the operating room. After giving intravenous dexamethasone 5 mg before anesthesia induction, all patients were sequentially given midazolam (0.03–0.05 mg/kg), sufentanil (0.3–0.5 μg/kg), etomidate (0.1–0.4 mg/kg), and rocuronium (0.6–1.0 mg/kg) to induce anesthesia, followed by insertion of a double-lumen bronchial tube for mechanical ventilation. A bispectral index value of 40–60 was maintained using continuous intravenous infusions of 50–100 μg/kg of propofol per minute and 0.1–1.0 μg/kg of remifentanil per minute; 0.03 mg/kg cisatracurium was injected intermittently to maintain proper muscle relaxation. Volume-controlled ventilation was performed to maintain an end-tidal carbon dioxide concentration between 35 and 45 mmHg. During the operation, single-lung ventilation was performed with a tidal volume of 6 mL/kg (predicted body weight) and 5 cmH_2_O PEEP. Before the end of the operation, both lungs were ventilated with an airway-positive pressure of 30 cmH_2_O for 15–30 s to promote lung re-expansion.

Patients were randomly assigned to one of four groups (Dex1, Dex2, Dex3 or Control group) following a 1:1:1:1 ratio using a computer-generated random number table. Dexmedetomidine (0.2 μg/kg/h, 0.4 μg/kg/h and 0.8 μg/kg/h) was given after induction until 30 min before the end of surgery in the Dex1, Dex2, and Dex3 groups, respectively; patients in the Control group were given the same dose of normal saline after induction until 30 min before the end of the surgery.

During surgery, the fluctuation range of blood pressure and heart rate was maintained within ±20% of the preoperative baseline value (defined as the average of three consecutive blood pressure values after entering the room). Norepinephrine (0.02–0.20 μg/kg/min) was administered when hypotension occurred (defined as a decrease in arterial blood pressure of more than 20% of the basic value). Nazardipine (0.2 mg) was administered if the patient had hypertension (defined as an increase in arterial blood pressure of more than 20% of the basic value). Atropine (0.3 mg) was administered if the patient had bradycardia (heart rate lower than 50 beats/min).

Each patient received intravenous ondansetron and propacetamol 15 min before the end of surgery. Postoperative analgesia was achieved with a patient-controlled analgesia pump (0.2 μg/kg hydromorphone, 15 mg tropisetron, and 360 mg ketorolac tromethamine in 150 mL normal saline). The background infusion rate was 2 mL per hour, the single dose was 1.5 mL per time, and the lockout time was 15 min.

### 2.3 Demographics and perioperative variables

The demographics and baseline measurements included age, body mass index (BMI), ASA classification, history of hypertension, diabetes, nonthoracic surgery, smoking, motion sickness, and PONV and preoperative anxiety score (Hamilton Anxiety Rating Scale). The intraoperative clinical variables included type of surgery, surgical site, duration of surgery and anesthesia, fluids administered, bleeding and urine output.

### 2.4 The outcomes

The primary outcome was the incidence of PONV during the postoperative 48 h. The secondary outcomes included the incidence of PONV and POV at different time points within 48 h after surgery (T1: PACU retention period; T2: PACU discharge to postoperative 12 h; T3: postoperative 12 h-postoperative 24 h; T4: postoperative 24 h-postoperative 48 h), PONV grade, POV grade and postoperative resting pain scores at T1-T4, the PONV_AUC_ (area under the curve of PONV grade over time) (Wengritzky et al., 2010; Damman et al., 2020; Mansour et al., 2022), intraoperative consumption of vasoactive drugs, Ramsay sedation score within Post Anesthesia Care Unit (PACU), PACU dwell time, PCA press frequency and sleep quality score within 48 h after surgery, incidence of remedial analgesics and antiemetics, time for first postoperative water intake, oral intake and ambulation, postoperative quality of recovery-15 (QOR-15) scores at 48 h after surgery and postoperative pulmonary complications during hospitalization. The intensity of PONV episode was graded as 0 (no nausea or vomiting), 1 (nausea noticed but no vomiting), 2 (vomiting noticed but no stomach contents spit out), or 3 (stomach contents spit out). The intensity of POV episode was graded as 0 (no vomiting), 1 (vomiting 1∼2 times), 2 (vomiting 3∼4 times), or 3 (vomiting > 5 times). Postoperative resting pain was measured using the visual analog scale (VAS), with “0” indicating no pain and “10” indicating unbearable, severe pain. Sleep quality was assessed using a numerical rating scale, with “0” indicating insomnia throughout the night, scores from 0 to 3 indicating severe insomnia, scores from 3 to 7 indicating moderate insomnia, scores above 7 indicating good sleep quality, and a score of 10 indicating excellent sleep quality.

### 2.5 Sample size

PASS 15 software was used to calculate the sample size. Since all the subjects were divided into the Dex1 group (weak intervention group), Dex2 group (medium intervention group), Dex3 group (strong intervention group), and Control group (blank control group), with the incidence of PONV during postoperative 48 h as the primary outcome, based on our pilot study results, the incidence rate of PONV in the Dex1 group was 40%, that in the Dex2 group was 30%, that in the Dex3 group was 40%, and that in the Control group was 75%. With a significance level (α) of 0.05, a power of 80%, and an effect size of 0.376, the calculated total sample size for the four groups using PASS 15 software is 148 patients. Considering a dropout rate of 15%, a total of 176 subjects were planned to be included in the study.

### 2.6 Statistical analysis

SPSS 23.0 software was used to perform statistical analyses. For continuous variables, depending on the normality of the distribution, data are expressed as the mean ± standard deviation or median and interquartile range. Group comparisons were performed using one-way ANOVA or the Kruskal‒Wallis test. Count data are presented as the percentage/composition ratio, and the χ2-square test or Fisher’s exact probabilities were used to compare the differences. The Bonferroni method was used for pairwise comparisons between the groups. Multivariate logistic regression analysis was used to screen for risk factors for PONV in female patients undergoing thoracoscopic radical lung cancer surgery. Differences were considered significant when the *p* values were <0.05.

## 3 Results

### 3.1 General characteristics

**FIGURE 1:**
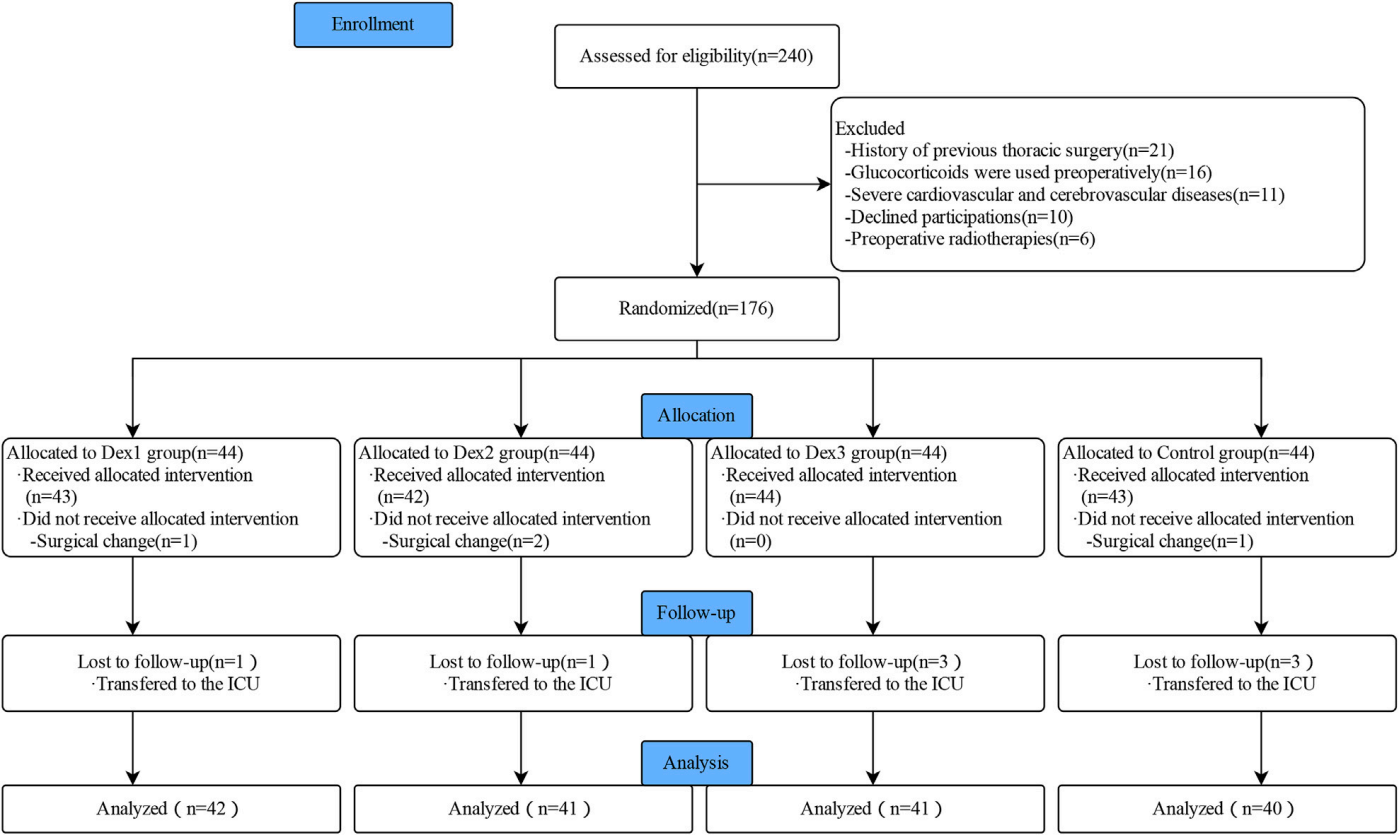
Flowchart of Consolidated Standards of Reporting Trials (CONSORT) describing patients’ progress throughout the study.

A total of 240 patients were initially enrolled in this study, and 164 patients were finally included in the statistics, including 42 patients in the Dex1 group, 41 patients in the Dex2 group, 41 patients in the Dex3 group, and 40 patients in the Control group (Figure 1). There were no differences in the comparison of the general characteristics among the four groups of patients (Table 1). There were no differences in the surgical site, intraoperative infusion volume, intraoperative bleeding and urine output, operation time, anesthesia time, intraoperative sufentanil dosage, intraoperative propofol dosage, or intraoperative atropine dosage among the four groups of patients. The consumption of remifentanil in the four groups was statistically significant (Dex1: 879.67 ± 258.98 μg vs. Dex2: 710.11 ± 232.55 μg vs. Dex3: 702.47 ± 249.63 μg vs. Control: 881.83 ± 226.60 μg, *p* < 0.001), and the Dex1 and Control groups were higher than the Dex2 group and Dex3 group. There was a statistically significant difference in the consumption of the vasoactive drug norepinephrine among the four groups during surgery [Dex1: 0 (0,0) µg vs. Dex2: 0 (0,0) µg vs. Dex3: 0 (0,70) µg vs. Control; 0 (0,0) µg, *p* = 0.012], and the consumption in the Dex3 group was higher than that in the Control group.

**TABLE 1:** Demographic data and perioperative characteristics.

	Dex1	Dex2	Dex3	Control	F/χ^2^	*p*
	(*n* = 42)	(*n* = 41)	(*n* = 41)	(*n* = 40)		
Age (year)	52.83 ± 9.38	53.61 ± 11.21	53.17 ± 8.30	51.63 ± 7.67	0.342	0.795
BMI (kg/m^2^)	23.55 ± 2.88	22.54 ± 3.54	24.35 ± 3.18	23.57 ± 3.41	2.128	0.099
ASA classification					3.534	0.759
Ⅰ	3 (7.14%)	3 (7.32%)	6 (14.63%)	2 (5.00%)		
Ⅱ	35 (83.33%)	33 (80.49%)	32 (78.05%)	32 (80.00%)		
Ⅲ	4 (9.52%)	5 (12.20%)	3 (7.32%)	6 (15.00%)		
History of hypertension					4.348	0.219
Yes	5 (11.90%)	8 (19.51%)	12 (29.27%)	6 (15.00%)		
No	37 (88.10%)	33 (80.49%)	29 (70.73%)	34 (85.00%)		
History of diabetes					0.726	0.896
Yes	4 (9.52%)	6 (14.63%)	5 (12.20%)	4 (10.00%)		
No	38 (90.48%)	35 (85.37%)	36 (87.80%)	36 (90.00%)		
History of non-thoracic surgery					1.910	0.598
Yes	18 (42.86%)	20 (48.78%)	19 (46.34%)	23 (57.50%)		
No	24 (57.14%)	21 (51.22%)	22 (53.66%)	17 (42.50%)		
History of smoking					2.200	0.473
Yes	1 (2.38%)	2 (4.88%)	4 (9.76%)	3 (7.50%)		
No	41 (97.62%)	39 (95.12%)	37 (90.24%)	37 (92.50%)		
History of motion sickness					0.637	0.903
Yes	8 (19.05%)	6 (14.63%)	8 (19.51%)	6 (15.00%)		
No	34 (80.95%)	35 (85.37%)	33 (80.49%)	34 (85.00%)		
History of PONV					0.875	0.865
Yes	3 (7.14%)	4 (9.76%)	5 (12.20%)	3 (7.50%)		
No	39 (92.86%)	37 (90.24%)	36 (87.80%)	37 (92.50%)		
Anxiety score	8.12 ± 4.10	9.34 ± 5.31	7.41 ± 4.51	7.30 ± 3.86	1.787	0.152
Surgical site					12.610	0.390
Upper lobe of the right lung	13 (30.95%)	17 (41.46%)	10 (24.39%)	12 (30.00%)		
Middle lobe of the right lung	1 (2.38%)	3 (7.32%)	1 (2.44%)	7 (17.50%)		
Lower lobe of the right lung	7 (16.67%)	7 (17.07%)	11 (26.83%)	6 (15.00%)		
Upper lobe of the left lung	13 (30.95%)	9 (21.95%)	13 (31.71%)	11 (27.50%)		
Lower lobe of the left lung	8 (19.05%)	5 (12.20%)	6 (14.63%)	4 (10.00%)		
Crystalloid solution (mL)	954.67 ± 201.63	1038.71 ± 331.59	966.12 ± 295.77	988.33 ± 333.90	0.657	0.579
Colloidal solution (mL)	0 (0,0)	0 (0,0)	0 (0,0)	0 (0,0)	0.842	0.844
Bleeding (mL)	36.07 ± 17.38	35.95 ± 21.42	33.39 ± 18.72	35.50 ± 22.95	0.157	0.925
Urine output (mL)	372.86 ± 47.64	362.20 ± 69.91	368.54 ± 81.56	363.20 ± 79.97	0.202	0.895
Surgical time (min)	165.76 ± 37.77	149.66 ± 42.68	144.51 ± 41.93	158.15 ± 39.03	2.231	0.087
Anesthesia time (min)	179.83 ± 36.52	165.63 ± 44.87	163.05 ± 43.81	173.00 ± 38.02	1.422	0.283
Sufentanil consumption (μg)	36.07 ± 7.49	35.95 ± 8.55	35.46 ± 9.11	35.50 ± 6.39	0.062	0.980
Remifentanil consumption (μg)	879.67 ± 258.98	710.11 ± 232.55^a^	702.47 ± 249.63^a^	881.85 ± 226.60^bc^	7.059	0.001
Propofol consumption (mg)	960.37 ± 294.00	839.54 ± 280.91	853.01 ± 330.91	956.43 ± 247.16	2.059	0.108
Norepinephrine consumption (µg)	0 (0,0)	0 (0,0)	0 (0,70)	0 (0,0)^c^	14.219	0.003
Atropine consumption (mg)	0 (0,0)	0 (0,0)	0 (0,0)	0 (0,0)	2.057	0.576

Values are presented as mean ± standard deviation or the number of patients (%). BMI, body mass index; ASA, American society of anesthesiologists. Compared to the Dex1 group, ^a^ *p* < 0.05; compared to the Dex2 group, ^b^ *p* < 0.05; compared to the Dex3 group, ^c^ *p* < 0.05.

### 3.2 Primary outcome

**FIGURE 2:**
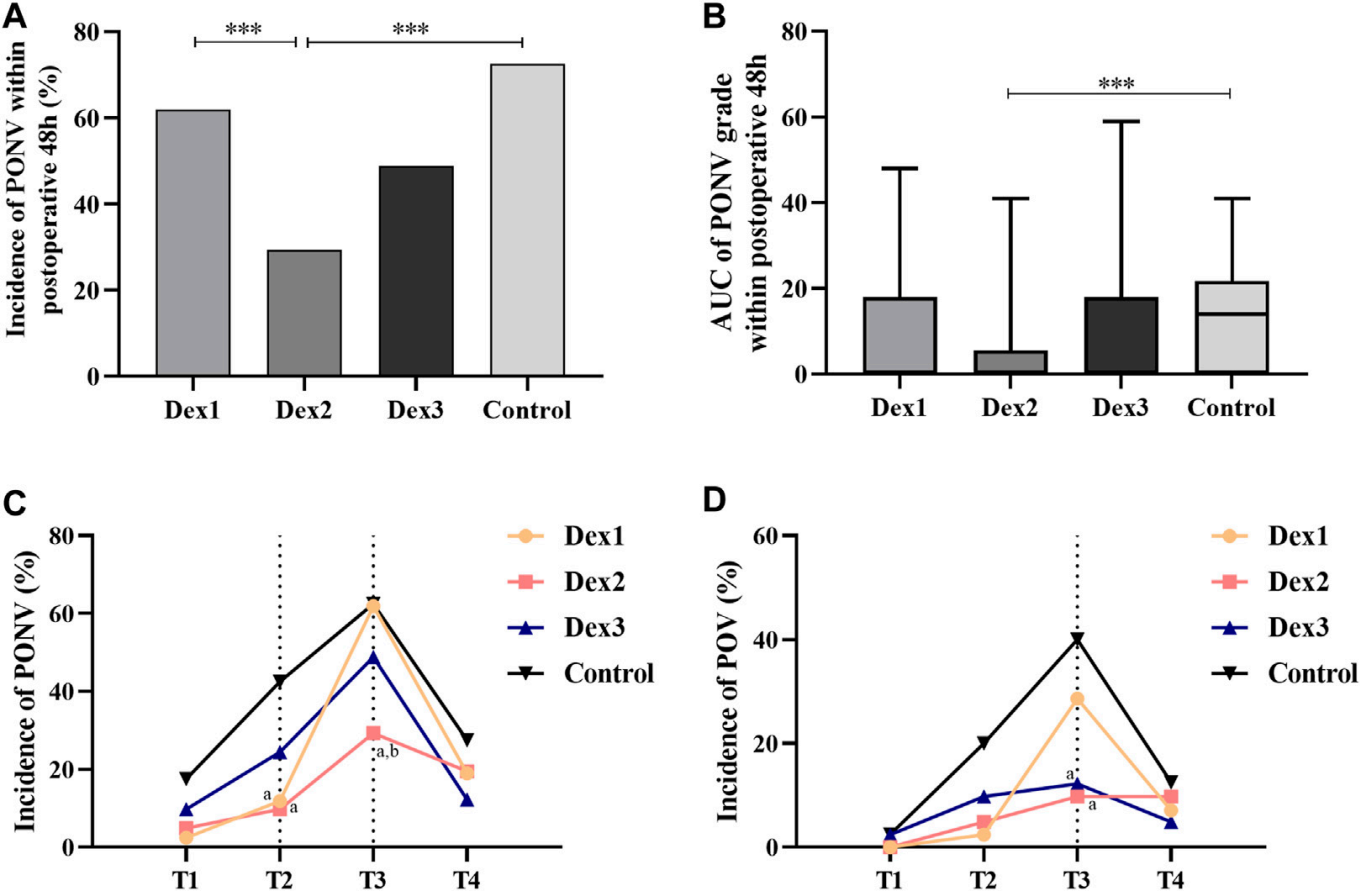
Comparison of PONV in all groups. Values are presented as the number of patients (%) or median and interquartile range. **(A)** The incidence of PONV within postoperative 48 h; **(B)** The AUC of PONV grade within postoperative 48 h; **(C)** The incidence of PONV at different T1-T4; **(D)** The incidence of POV at different T1-T4. ***p < 0.005; compared to the Control group, ^a^ *p* < 0.05; compared to the Dex1 group, ^b^ *p* < 0.05.

There was a significant difference in the incidence of PONV among the four groups (Dex1: 61.90% vs. Dex2: 29.27% vs. Dex3: 48.78% vs. Control: 72.50%, *p* < 0.001) (Figure 2A). Dexmedetomidine could reduce the incidence of PONV. The incidence of PONV in the Dex2 group (29.27%) was lower than that in the Dex1 group (61.90%) and Control group (72.50%).

### 3.3 Secondary outcomes

#### 3.3.1 Incidence of PONV at four time periods

There was a significant difference in the incidence of PONV among the four groups at T2 (Dex1: 11.90% vs. Dex2: 9.76% vs. Dex3: 24.39% vs. Control: 42.50%, *p* = 0.002) (Figure 2C), and the incidence of PONV in the Dex1 and Dex2 groups was lower than that in the Control group. There was a statistically significant difference in the incidence of PONV among the four groups at T3 (Dex1: 61.90% vs. Dex2: 29.27% vs. Dex3: 48.78% vs. Control: 62.50%, *p* = 0.007) (Figure 2C), and the incidence of PONV in the Dex2 group was lower than that in the Dex1 and Control groups.

#### 3.3.2 PONV_AUC_ and PONV grade

There were statistically significant differences in PONV_AUC_ among the four groups (Dex1: 0.0 (0.0, 18.0) vs. Dex2: 0.0 (0.0, 0.0) vs. Dex3: 0.0 (0.0, 18.0) vs. Control: 14.0 (0.0, 20.5), *p* = 0.004), and PONV_AUC_ was lower in the Dex2 group than in the Control group (Figure 2B; Table 2).

**TABLE 2:** AUC of PONV grade over times within 48 h after surgery.

Time	Dex1	Dex2	Dex3	Control	H	P
	(*n* = 42)	(*n* = 41)	(*n* = 41)	(*n* = 40)		
2H	0.0 (0.0,0.0)	0.0 (0.0,0.0)	0.0 (0.0,0.0)	0.0 (0.0,0.0)	5.428	0.143
12H	0.0 (0.0,0.0)	0.0 (0.0,0.0)	0.0 (0.0,0.0)	0.0 (0.0,0.0)	5.150	0.161
24H	0.0 (0.0,1.0)	0.0 (0.0,0.0)	0.0 (0.0,1.0)	0.0 (0.0,1.0)^b^	10.260	0.016
48H	0.0 (0.0,0.0)	0.0 (0.0,0.0)	0.0 (0.0,0.0)	0.0 (0.0,0.0)	4.578	0.205
AUC	0.0 (0.0,18.0)	0.0 (0.0,0.0)	0.0 (0.0,18.0)	14.0 (0.0,20.5)^b^	13.403	0.004

Values are presented as median and interquartile range. The terms 2H, 12H, 24H, and 48H correspond to the PONV grades at the respective postoperative time points of 2 h, 12 h, 24 h, and 48 h. AUC, area under the curve of PONV, grade over time. Compared to the Dex1 group, ^a^ *p* < 0.05; compared to the Dex2 group, ^b^ *p* < 0.05; compared to the Dex3 group, ^c^ *p* < 0.05.

**FIGURE 3:**
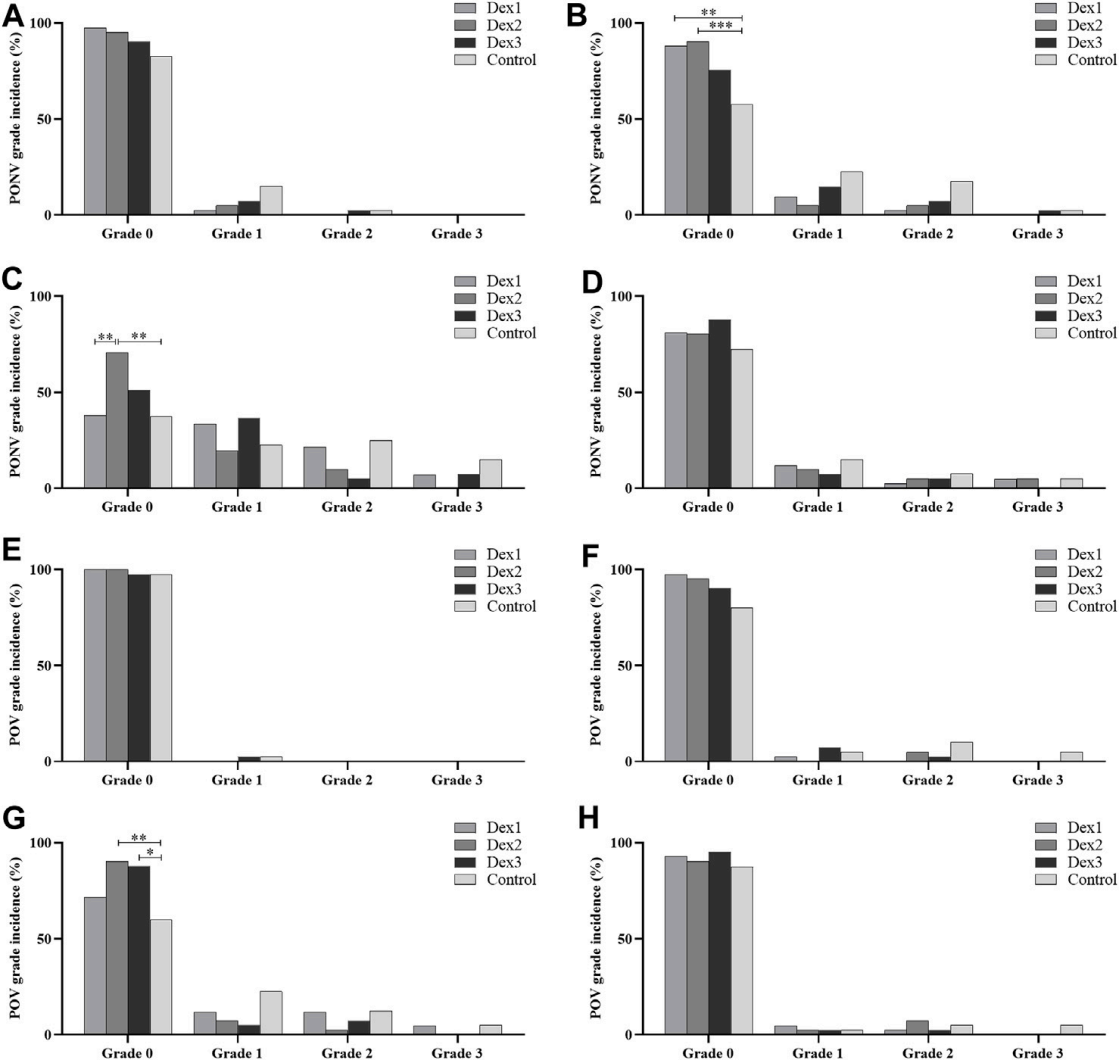
Comparison of PONV and POV grades in all groups. Values are presented as the number of patients (%). **(A–D)**: incidence of PONV grades for the respective time periods from T1 to T4; **(E–H)**: incidence of POV grades for the respective time periods from T1 to T4. *p < 0.05; **p < 0.01; ***p < 0.005.

There were statistically significant differences in the proportion of grade 0 PONV at T2 among the four groups (Dex1: 88.10% vs. Dex2: 90.24% vs. Dex3: 75.61% vs. Control: 57.50%, *p* = 0.002). The proportions in the Dex1 and Dex2 groups were higher than that in the Control group (Figure 3B). There were no differences in the proportions of grade 1 PONV (Dex1: 9.48% vs. Dex2: 4.88% vs. Dex3: 14.63% vs. Control: 22.50%, *p* = 0.111), grade 2 PONV (Dex1: 2.38% vs. Dex2: 4.88% vs. Dex3: 7.32% vs. Control: 17.50%, *p* = 0.097) and grade 3 PONV (Dex1: 0.00% vs. Dex2: 0.00% vs. Dex3: 2.44% vs. Control: 2.50%, *p* = 0.491) at T2 among the four groups.

There were differences in the proportion of grade 0 PONV at T3 among the four groups (Dex1: 38.10% vs. Dex2: 70.73% vs. Dex3: 51.22% vs. Control: 37.50%, *p* = 0.003) (Figure 3C). The proportion in the Dex2 group was higher than that in the Dex1 and Control groups. There were differences in the proportion of grade 2 PONV at T3 among the four groups (Dex1: 21.429% vs. Dex2: 9.756% vs. Dex3: 4.878% vs. Control: 25.00%, *p* = 0.033). There were no statistically significant differences in the proportions of grade 1 PONV (Dex1: 33.33% vs. Dex2: 19.05% vs. Dex3: 36.59% vs. Control: 22.50%, *p* = 0.227) and grade 3 PONV (Dex1: 7.14% vs. Dex2: 0.00% vs. Dex3: 7.32% vs. Control: 15.00%, *p* = 0.061) at T3 among the four groups.

#### 3.3.3 POV incidence and grade

The difference in the incidence of POV at T3 among the four groups of patients was statistically significant (Dex1: 28.57% vs. Dex2: 9.76% vs. Dex3: 12.20% vs. Control: 40.00%, *p* = 0.003), and the incidence in the Dex2 and Dex3 groups was lower than that in the Control group (Figure 2D). At T1, T2, and T4 period, there was no difference in the incidence of POV or the POV grade among the four groups (Figure 2D; Figures 3E–H).

#### 3.3.4 Postoperative pain

**FIGURE 4:**
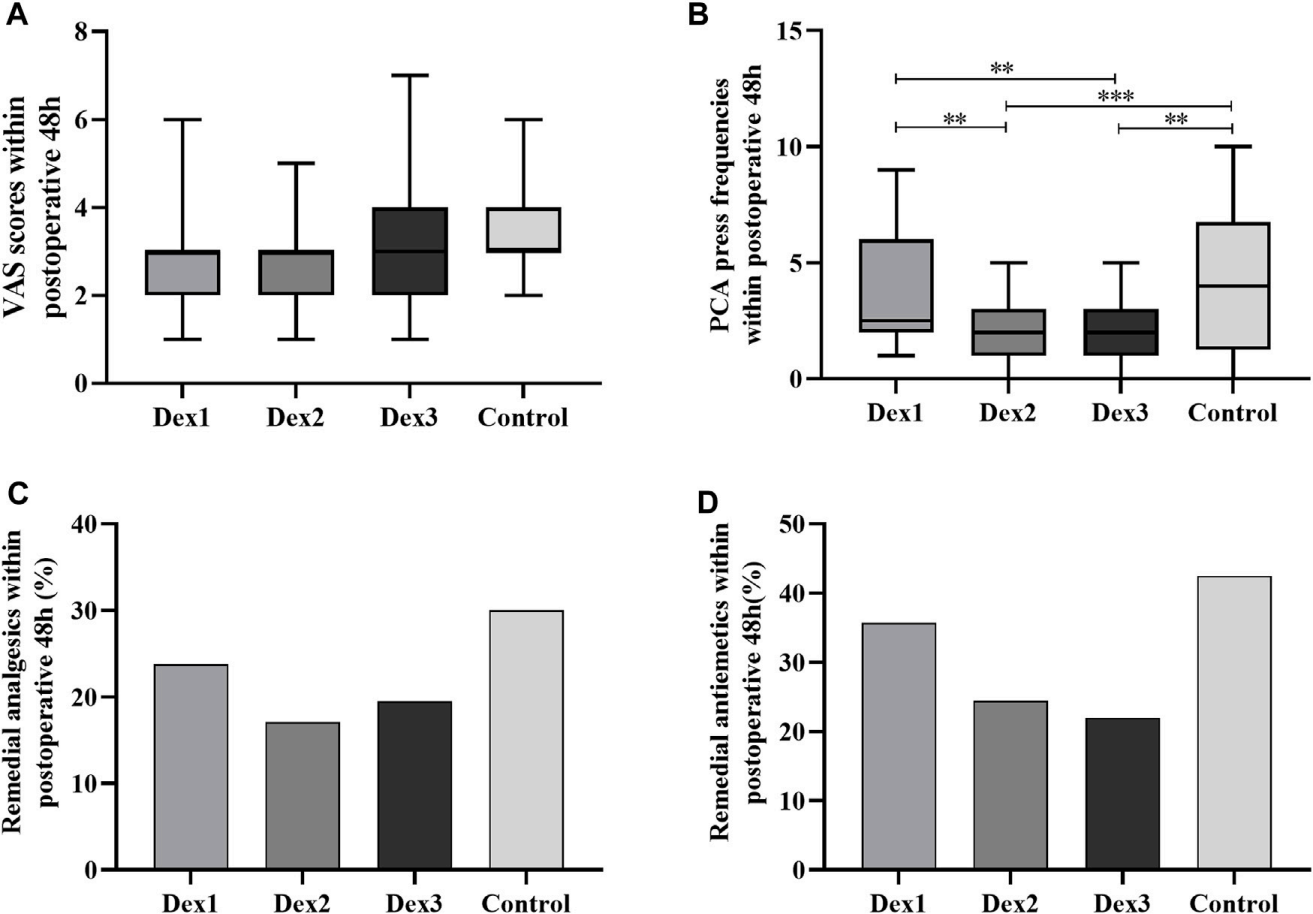
Comparison of postoperative pain in all groups. Values are presented as the number of patients (%) or median and interquartile range. **(A)** The VAS Scores within postoperative 48 h; **(B)** The PCA press frequencies within postoperative 48 h; **(C)** The remedial analgesics within postoperative 48 h; **(D)** The remedial antiemetics within postoperative 48 h. PCA, patient-controlled analgesia. *p < 0.05; **p < 0.01; ***p < 0.005.

There was no difference of the VAS score and incidence of remedial analgesics within postoperative 48 h among the four groups (Figures 4A, C). There was a difference in the PCA press frequency within 48 h after surgery among the four groups (Dex1: 2.50 (2.00–6.00) vs. Dex2: 2.00 (1.00–3.00) vs. Dex3: 2.00 (1.00–2.50) vs. Control: 4.00 (1.25–6.75), *p* < 0.001) (Figure 4B). The Dex2 and Dex3 groups had lower levels than the Control group and the Dex1 group.

#### 3.3.5 Postoperative recovery

**FIGURE 5:**
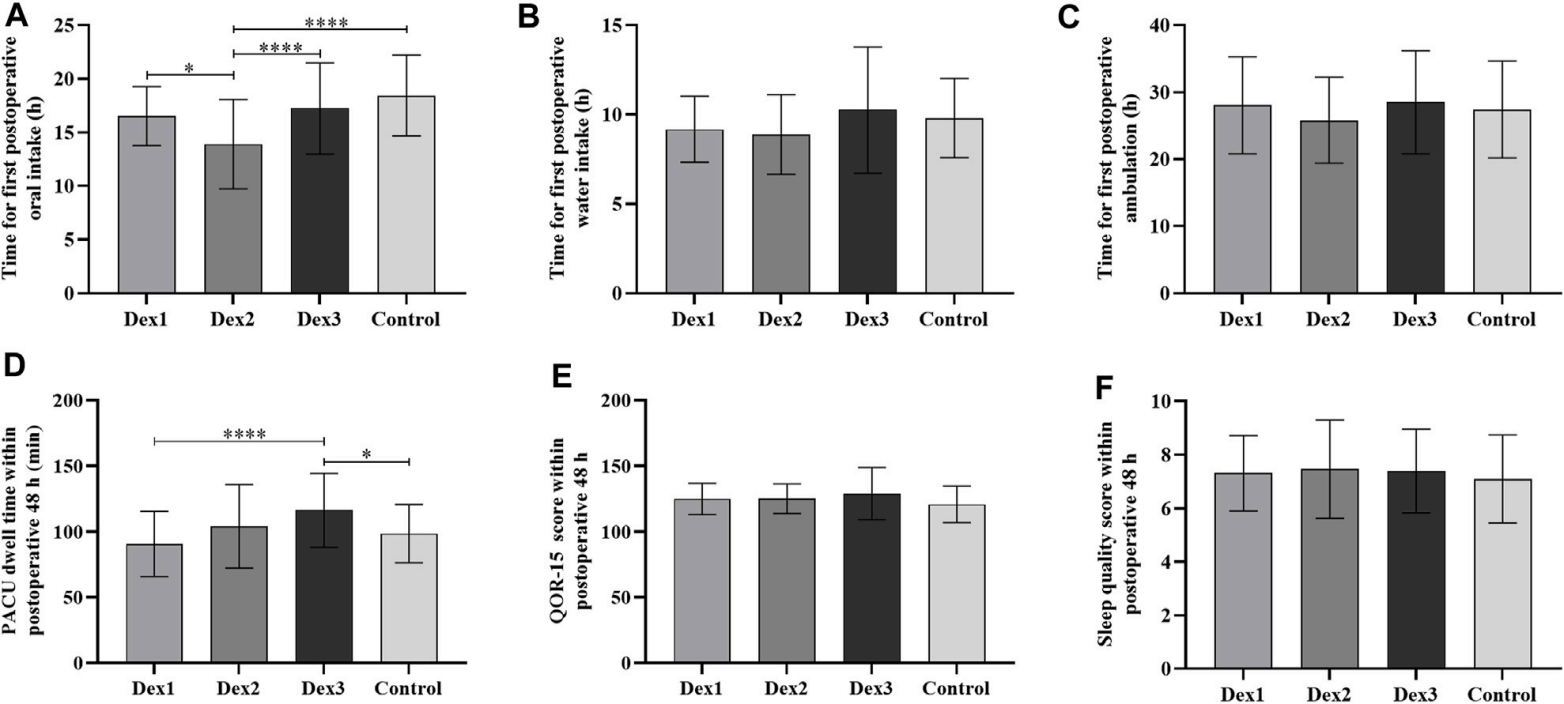
Comparison of postoperative recovery in all groups. Values are presented as mean ± standard deviation. **(A)** The time for first postoperative oral intake; **(B)** The time for first postoperative water intake; **(C)** The time for first postoperative ambulation; **(D)** The PACU dwell time within postoperative 48 h; **(E)** The QOR-15 score within postoperative 48 h; **(F)** The sleep quality score within postoperative 48 h. QOR-15, postoperative quality of recovery-15. *p < 0.05; ****p < 0.001.

There was a difference in PACU dwell time among the four groups (Dex1: 90.64 ± 24.79 vs. Dex2: 104.02 ± 31.83 vs. Dex3: 115.23 ± 27.86 vs. Control: 98.45 ± 22.17, *p* < 0.001), and that in the Dex3 group was higher than that in the Dex1 and Control groups (Figure 5D).

The time to first postoperative oral intake among the four groups was different (Dex1: 16.52 ± 2.74 h vs. Dex2: 13.90 ± 4.17 h vs. Dex3: 17.22 ± 4.25 h vs. Control: 18.43 ± 3.78 h, *p* < 0.001), and the Dex2 group was shorter than the other three groups (Figure 5A).

There was no difference among the four groups in the time to first postoperative water intake and ambulation, quality of recovery-15 (QOR-15) score, sleep score, use of remedial analgesics and antiemetics, or postoperative pulmonary complications (Figures 4, 5; Table 3).

**TABLE 3:** Postoperative pulmonary complications.

	Dex1	Dex2	Dex3	Control	χ^2^	P
	(*n* = 42)	(*n* = 41)	(*n* = 41)	(*n* = 40)		
Pulmonary complications					9.770	0.894
None	8 (19.05%)	5 (12.20%)	4 (9.76%)	4 (10.00%)	2.103	0.548
Inflammation possible	18 (42.86%)	21 (51.22%)	23 (56.10%)	22 (55.00%)	1.795	0.629
Pleural effusion	1 (2.38%)	0 (0.00%)	1 (2.44%)	1 (5.00%)	1.020	0.902
Inflammation possible + Pleural effusion	14 (33.33%)	15 (36.59%)	11 (26.83%)	11 (27.50%)	1.264	0.746
Inflammation possible + Pneumothorax	1 (2.38%)	0 (0.00%)	1 (2.44%)	2 (5.00%)	2.095	0.471
Inflammation possible + Pleural effusion + Pneumothorax	0 (0.00%)	0 (0.00%)	1 (2.44%)	0 (0.00%)	2.823	0.744

Values are presented as the number of patients (%).

### 3.4 Risk factors and protective factors for PONV

**FIGURE 6:**
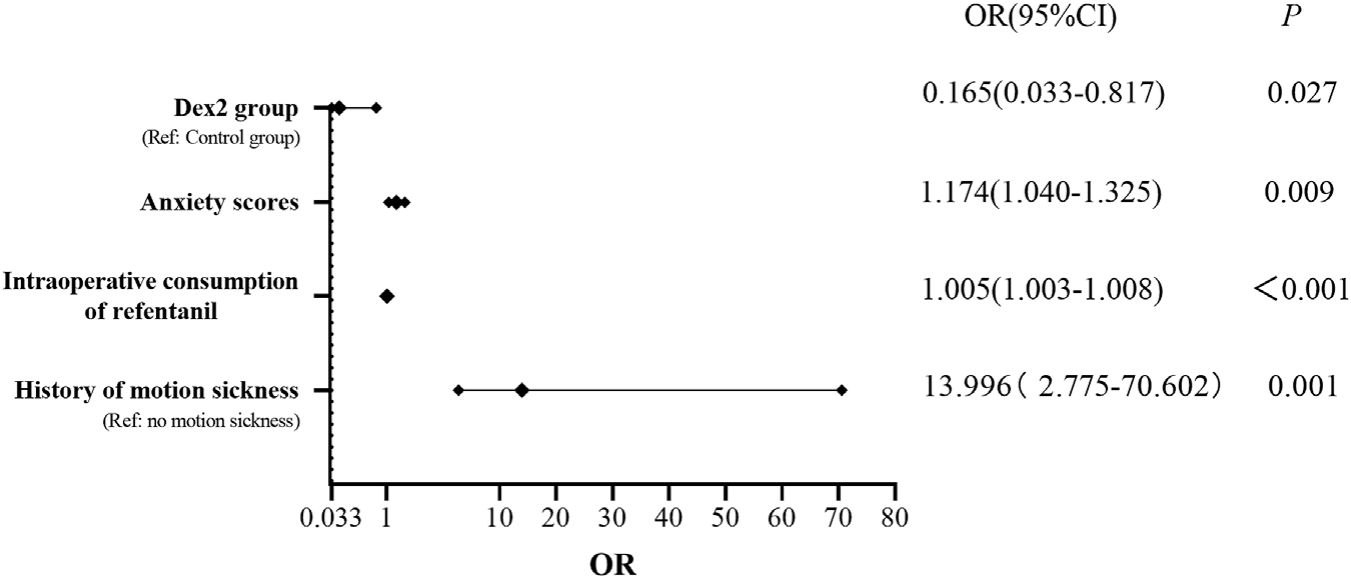
Risk factors and protective factors for PONV Dex2 group was a protective factor for PONV, while the preoperative anxiety score, intraoperative consumption of remifentanil and history of motion sickness were risk factors for PONV. OR, odds ratio; CI, confidence interval.

The univariate logistic regression analysis of possible PONV risk or protective factors variables (dexmedetomidine, age, height, weight, BMI, ASA classification, history of smoking, history of PONV and motion sickness, nonthoracic surgery history, history of hypertension, history of diabetes, preoperative anxiety score, surgical site, intraoperative bleeding and urine output, duration of surgery and anesthesia, intraoperative consumption of propofol, intraoperative consumption of sufentanil, intraoperative consumption of remifentanil, crystalloid infusion volume, colloid infusion volume, intraoperative consumption of vasopressors, PACU dwell time, PCA press frequency within 48 h, sleep quality score within 48 h, consumption of remedial analgesics, consumption of remedial antiemetics, QOR-15 score and postoperative pulmonary complications) showed that the different doses of intraoperative dexmedetomidine, duration of surgery, duration of anesthesia, intraoperative consumption of sufentanil, intraoperative consumption of remifentanil, preoperative anxiety score and history of motion sickness were statistically significant in relation to the incidence of PONV. The multivariate logistic regression analysis showed that the Dex2 group was a protective factor for PONV, while the preoperative anxiety score, intraoperative consumption of remifentanil and history of motion sickness were risk factors for PONV (Figure 6).

## 4 Discussion

To the best of our knowledge, our study is the first to reveal the dynamic effect of different dosages of dexmedetomidine on PONV in female patients undergoing thoracoscopic radical lung cancer surgery. In general, PONV mainly occurred during the first 24 h after surgery. The protective effect of dexmedetomidine on PONV in female patients was verified in our study. Dexmedetomidine (0.4 μg/kg) was preferable to the other two Dex groups. Our study also found that the PONV grade, PONV_AUC_ and POV were improved by dexmedetomidine. In addition, PCA frequencies and the time to first postoperative oral intake were also affected by dexmedetomidine. Finally, based on our results, we constructed a regression model for the risk factors for PONV. Dexmedetomidine (0.4 μg/kg/h) was a protective factor against PONV.

Research by Wang et al. demonstrated that 0.5 μg/kg/h dexmedetomidine during thoracoscopic lung lobectomy reduced the incidence of PONV within 6 h postoperatively compared to saline infusion (Wang et al., 2016). Mohta et al. also found that 0.6 μg/kg dexmedetomidine alleviated postoperative pain and maintained hemodynamic stability on laparoscopic cholecystectomy with a surgical duration of approximately 1 h (Mohta et al., 2016). Tufanogullari et al. conducted a study on reduced-port laparoscopic surgery and found that intravenous infusion of dexmedetomidine at doses of 0.2–0.8 μg/kg/h reduced the consumption of fentanyl, postoperative antiemetics, and the length of stay in the PACU (Tufanogullari et al., 2008). Based on previous studies and our preliminary experiments, this study ultimately selected three different doses of 0.2 μg/kg/h, 0.4 μg/kg/h, and 0.8 μg/kg/h for investigation.

Studies have shown that the risk of PONV decreases with increasing age in adults (Eberhart et al., 2000; Apfel et al., 2012a). The subjects of this study are restricted to adult women who are a high-risk population for PONV. Patients aged 18–65 years were selected as the study population, while more elderly patients and children were excluded. The average age of the four groups of patients ranged from 51 to 54 years, aiming to minimize the impact of age on the experimental results. Furthermore, there were no differences in smoking history, history of PONV, history of motion sickness, or other general characteristics. To reduce the influence of anesthesia factors on the results of this study, this study uniformly used total intravenous general anesthesia (Visser et al., 2001) and no volatile anesthetics, which increased the comparability of this study. Studies have indicated that serotonin receptor antagonists (such as ondansetron) and/or corticosteroids (such as dexamethasone) are the preferred medications for preventing vomiting (Golembiewski and Tokumaru, 2006; Sridharan and Sivaramakrishnan, 2019). Therefore, to maximize the protection of patient rights and interests, we administered 5 mg of dexamethasone to each patient before induction and a prophylactic dose of 5 mg of ondansetron 15 min before the end of surgery.

The incidence of PONV is a dynamic process; therefore, based on previous literature and the preliminary experiment, we selected four time points within 48 h postoperatively for evaluation. Luo’s study showed that after administering a loading dose of 1 μg/kg of dexmedetomidine during surgery, followed by a maintenance dose of 0.6 μg/kg/h of dexmedetomidine, it can effectively reduce the pain scores at 1 h, 2 h, and 4 h after submucosal dissection of gastric endoscopy and decrease the severity of PONV (Luo et al., 2023). Lee’s study showed that 0.4 μg/kg/h dexmedetomidine reduced the incidence of PONV and alleviated postoperative pain in patients undergoing laparoscopic hysterectomy within 24 h postoperatively (Lee et al., 2022). Similar to the above experimental results, our study revealed that 0.4 μg/kg/h dexmedetomidine could effectively reduce the incidence of PONV. In addition, compared with the method of simply selecting a fixed postoperative time point, the area under the curve can reflect the dynamic trend of different postoperative time points. The PONV_AUC_ can reflect the severity of PONV over a period of time. Our study shows that the PONV_AUC_ was lower in the Dex2 group than in the Control group, and dexmedetomidine could improve the PONV_AUC_ for a period of time after surgery.

Our study demonstrates that dexmedetomidine can significantly reduce the incidence of PONV. We speculate that the reason partially lies in its analgesic properties (Afonso and Reis, 2012; Chima et al., 2022). According to the studies conducted by Zhang and Kaye (Kaye et al., 2020; Zhang et al., 2022), intraoperative administration of dexmedetomidine can alleviate postoperative pain. Pain is an independent risk factor for the incidence of PONV. Our study shows dexmedetomidine reduces the PCA press frequencies through its synergistic analgesic effect, as the latter has been identified as a risk factor for PONV(Mauermann et al., 2019). The PCA press frequency in the treatment group was lower than that in the Control group. Therefore, we speculate that dexmedetomidine reduces the incidence of PONV through its synergistic analgesic effect by reducing the number of postoperative PCA presses.

Compared to the Dex1 and Dex3 groups, the Dex2 group showed significant efficacy in preventing and treating PONV and POV. Patients in the Dex3 group, who received a higher dose of dexmedetomidine, had a significantly longer dwell time in the PACU compared to the Dex1 group (low-dose dexmedetomidine) and the Control group. This is unfavorable for patient recovery and reduces the efficiency of the PACU. According to previous studies (Choi et al., 2017), the use of a loading dose of 1 μg/kg followed by a larger dose of 0.3–0.5 μg/kg/h of dexmedetomidine during surgery resulted in delayed recovery during the initial few hours after extubation. In addition, side effects associated with dexmedetomidine mainly include hypotension and bradycardia (Weerink et al., 2017). The dose of the vasoactive drug norepinephrine used in the Dex3 group was significantly higher than that in the Control group, suggesting that 0.8 μg/kg/h dexmedetomidine may have contributed to intraoperative hemodynamic instability.

Comprehensive optimization of the perioperative period based on evidence-based medicine aims to reduce surgical stress and complications and achieve rapid patient recovery (Simpson et al., 2019). Early oral intake and early mobilization after surgery are important components of enhanced recovery after surgery (ERAS). Studies have shown that early oral intake can maintain the intestinal mucosa, nourish the intestines, promote portal venous circulation, and accelerate gastrointestinal motility recovery (Terashima, 2014). Our study demonstrated that the time for the first postoperative oral intake in the Dex2 group was shorter than that in the other groups. This may be attributed to the good preventive effect of dexmedetomidine at a dose of 0.4 μg/kg/h on PONV and its analgesic action, which helps facilitate early postoperative diet and accelerate early patient recovery.

Our study further analyzed the risk factors for PONV through regression analysis. The results showed that intraoperative infusion of 0.4 μg/kg/h dexmedetomidine was a protective factor against PONV. Preoperative anxiety scores, intraoperative consumption of remifentanil, and history of motion sickness were found to influence the incidence of PONV, serving as risk factors for PONV, which is consistent with previous research findings. Laufenberg-Feldmann et al. found a close correlation between preoperative anxiety and PONV, with preoperative anxiety increasing the odds ratio of PONV by fivefold in nonsmoking women, suggesting that preoperative anxiety may be an important indicator for predicting PONV (Laufenberg-Feldmann et al., 2019). Roh et al. proposed that this phenomenon may be attributed to increased sympathetic nervous system activity in anxious patients, leading to increased release of catecholamines (Roh et al., 2014). Additionally, anxious patients tend to swallow excessive air, increasing gastric capacity, which may be associated with an increased incidence of POV. Opioid drugs can inhibit the release of acetylcholine in the mesenteric plexus and stimulate μ receptors, thereby reducing muscle tone and motility and triggering PONV through the serotonergic signaling pathway (de Boer et al., 2017). A history of motion sickness has been identified as a risk factor for predicting PONV, although the specific mechanisms are unclear and may be related to abnormal sensitivity of the vestibular organ (Momeni et al., 2006).

### 4.1 Limitations

This study has several limitations. First, this trial is a single-center study, and more multicenter studies with larger sample sizes are needed to verify the results. Second, this trial did not further distinguish age gradients and did not consider the difference in the incidence of PONV between pre- and postmenopausal populations (Sener et al., 2005). Third, this trial only used dexmedetomidine intraoperatively, and the effect of postoperative use of dexmedetomidine on the prevention and treatment of PONV or combined use of dexmedetomidine during and after surgery is unknown. Fourth, this trial did not detect the related neurotransmitters, and the mechanism of dexmedetomidine on reducing PONV needs further study.

## 5 Conclusion

For female patients undergoing thoracoscopic radical lung cancer surgery, infusion of dexmedetomidine during total intravenous general anesthesia can reduce the incidence of PONV, reduce perioperative opioid use, improve postoperative analgesic effect, shorten the time for first postoperative oral intake, and promote early recovery of patients after surgery. Compared with the other two dosages, 0.4 μg/kg/h dexmedetomidine is preferable.

## Data Availability

The raw data supporting the conclusions of this article will be made available by the authors, without undue reservation.
